# Quantitative Anatomy of the Growing Lungs in the Human Fetus

**DOI:** 10.1155/2015/362781

**Published:** 2015-08-27

**Authors:** Michał Szpinda, Waldemar Siedlaczek, Anna Szpinda, Alina Woźniak, Celestyna Mila-Kierzenkowska, Mateusz Badura

**Affiliations:** ^1^Department of Normal Anatomy, Collegium Medicum of Nicolaus Copernicus University, Łukasiewicza 1 Street, 85-821 Bydgoszcz, Poland; ^2^Department of Medical Biology, Collegium Medicum of Nicolaus Copernicus University, Karlowicza 24 Street, 85-092 Bydgoszcz, Poland

## Abstract

Using anatomical, digital, and statistical methods we examined the three-dimensional growth of the lungs in 67 human fetuses aged 16–25 weeks. The lung dimensions revealed no sex differences. The transverse and sagittal diameters and the base circumference were greater in the right lungs while the lengths of anterior and posterior margins and the lung height were greater in the left lungs. The best-fit curves for all the lung parameters were natural logarithmic models. The transverse-to-sagittal diameter ratio remained stable and averaged 0.56 ± 0.08 and 0.52 ± 0.08 for the right and left lungs, respectively. For the right and left lungs, the transverse diameter-to-height ratio significantly increased from 0.74 ± 0.09 to 0.92 ± 0.08 and from 0.56 ± 0.07 to 0.79 ± 0.09, respectively. The sagittal diameter-to-height ratio significantly increased from 1.41 ± 0.23 to 1.66 ± 0.18 in the right lung, and from 1.27 ± 0.17 to 1.48 ± 0.22 in the left lung. In the fetal lungs, their proportionate increase in transverse and sagittal diameters considerably accelerates with relation to the lung height. The lung dimensions in the fetus are relevant in the evaluation of the normative pulmonary growth and the diagnosis of pulmonary hypoplasia.

## 1. Introduction

Lung growth determination indubitably comprises the most life-threatening constituent of the prenatal assessment [1]. Modern* in utero *diagnostic imaging (three-dimensional ultrasound, ultrafast MRI) enables clinicians to reliably detect congenital respiratory malformations [2, 3] and evaluate both maturation and well-being of the fetus [4, 5]. Antenatal lung growth and maturation are becoming increasingly relevant in determining survival and outcomes in both preterm newborns and neonates affected by pulmonary hypoplasia. Pulmonary hypoplasia appears secondary to many pathological conditions that hamper normal development of the fetal lungs [6–8]. It is noteworthy that apposite assessment of fetal development is mainly grounded in morphologic analysis and biometric measurements [9, 10].

To date however, apart from pulmonary volumetric patterns [6, 9–14], no nomograms on other lung dimensions in the human fetus have been computed. Therefore, in the present study we aimed to focus on the following:

- reference intervals for lung dimensions (transverse and sagittal diameters, base circumference, height, lengths of anterior, and posterior margins) at successive gestational ages (age-specific reference intervals),
- possible sex and laterality differences,
- the optimal growth curves against gestational age for the six aforementioned parameters,
- the relative growth of either lung (transverse-to-sagittal diameter ratio, transverse diameter-to-height ratio, and sagittal diameter-to-height ratio).

## 2. Materials and Methods

The present study was performed in Department of Anatomy of the Ludwik Rydygier Collegium Medicum in Bydgoszcz. The sample encompassed 67 autopsied human fetuses, comprising 35 males and 32 females of White racial origin, derived from spontaneous abortions or stillbirths in the years 1989–1999. First of all, the sample was constructed by elimination of fetuses from diabetic or multiple gravidities and specimens affected by innate and chromosomal abnormalities or intrauterine growth restriction. Legitimate and ethical dilemmas were sanctioned by the University Research Ethics Committee (KB 190/2011). After evaluating the fetal crown-rump length (gestational age), known date of the beginning of the last maternal menstrual period (amenorrhea age), and the five fetal anthropometric measurements (head circumference, biparietal diameter, occipitofrontal diameter, abdominal circumference, and femur length) assessed by early second-trimester ultrasound scan (ultrasound age), the fetal age of 16–25 weeks (Table 1) was fine-tuned [9, 15].

### 2.1. Anatomical Method

After having been submerged in 10% neutral buffered formalin solution for 12–24 months, the fetuses through sternotomy were anatomically dissected; then the lungs were cut off at their hila and removed out of the thoracic cavity. Since no lung malformations were macroscopically perceived in the individuals studied, the sample could rightly be considered normal.

### 2.2. Digital Image Analysis

**Figure 1:**
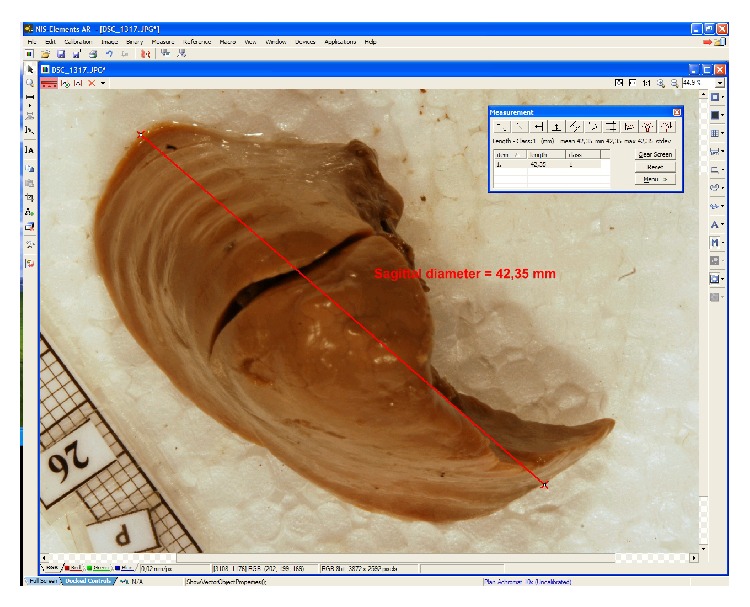
A screen of digital image analysis of NIS Elements AR 3.0 (Nikon) while assessing the sagittal diameter of the right lung.

Every isolated lung with a millimeter scale was positioned perpendicularly to the optical lens axis, recorded in superior, inferior (diaphragmatic), medial (mediastinal), and lateral (sternocostal) projections using NIKON D200 camera (with Micro-Nikkor AF-S 60 mm f/2.8 G ED lens), digitalized to TIFF images, and quantitatively assessed (Figure 1) with the use of digital image analysis. In the present study a valid objective automatic software package, that is, NIS Elements AR (Advanced Research) 3.0 (Nikon) was used for measuring the selected pulmonary dimensions in the fetus, with the greatest accuracy to the nearest 0.01 mm. As an optimized digital-image analysis system for advanced research applications, NIS-Elements AR 3.0 offers, among other things, flawlessly automatic six-dimensional (*X*, *Y*, *Z*, wavelength, *T*, multistage points) image acquisition, sophisticated image processing, peripheral device control, and data analysis.

**Figure 2:**
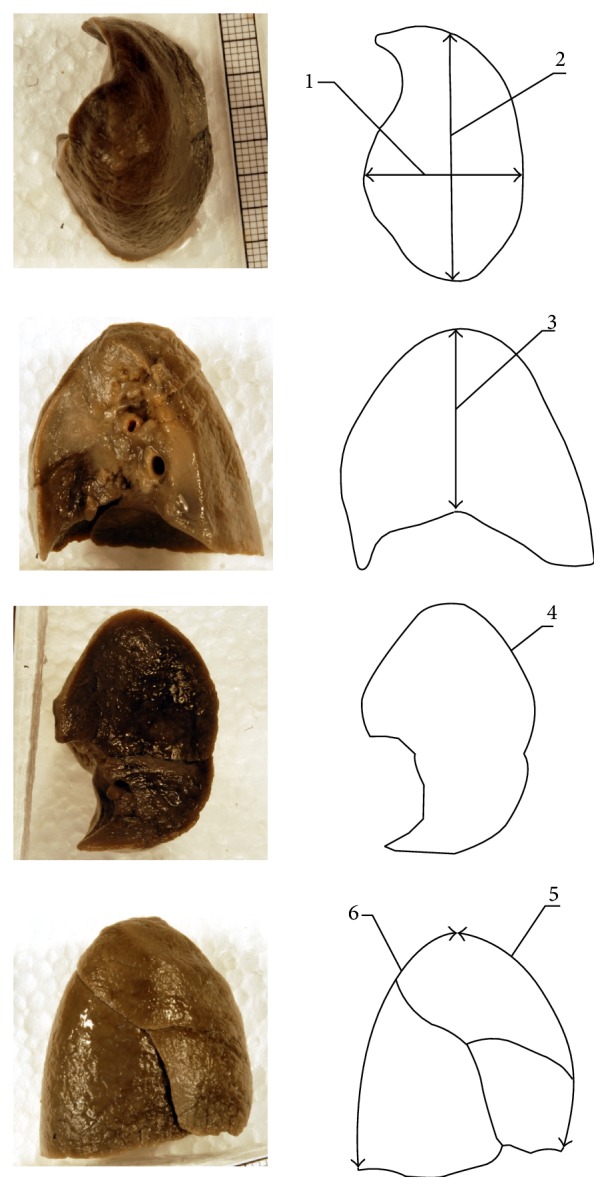
Measurements of the pulmonary parameters studied (with relation to the right lung): (1) transverse diameter, (2) sagittal diameter, (3) height, (4) base circumference, (5) length of the anterior margin, and (6) length of the posterior margin.

In every fetus for the right and left lungs the following six (Figure 2) independent measurements (1–6) in mm and three calculations (7–9) were done:

1. transverse diameter of the lung, corresponding to the greatest horizontal distance of the lung from lateral to medial surface, measured in its superior projection,
2. sagittal diameter of the lung, corresponding to the greatest horizontal distance of the lung from anterior to posterior margin, measured in its superior projection,
3. height of the lung, measured in its mediastinal projection,
4. base circumference, measured in its diaphragmatic projection,
5. length of the lung anterior margin, measured from the lung top to its base in its sternocostal projection,
6. length of the lung posterior margin, measured from the lung top to its base in its sternocostal projection,
7. transverse-to-sagittal diameter ratio,
8. transverse diameter-to-height ratio,
9. sagittal diameter-to height ratio.

### 2.3. Statistical Analysis

In a constant effort to minimize measurement and observer bias, all measurements were performed by one researcher (W.S.). Each measurement was done three times under the same settings but at diverse times and then averaged. The intraobserver variation between the reiterated measurements was evaluated by ANOVA for repeated measurements and post hoc RIR Tukey test. The numerical data were verified for normality of distribution (the Kolmogorov-Smirnov test) and homogeneity of variance (Levene's test). The fetuses studied were collected into 10 one-week intervals inadequately dispersed with fetal age. Since 3 fetuses were included in the gestational age of 25 weeks and even 2 fetuses in the gestational age of 16 weeks, which clearly did not represent adequate samples for statistical analysis, the first three intervals of 16–18 weeks (*n* = 20), the consecutive three intervals of 19–21 weeks (*n* = 27), and the last four intervals of 22–25 weeks (*n* = 20) were aggregated. The statistical analysis was started by assessing the probability of appearance of statistically significant differences in values with relation to sex (Student *t*-test for unpaired variables) and laterality (Student *t*-test for paired variables). In order to examine sex differences, at first we tested differences between the following three age groups, 16–18, 19–21, and 22–25 weeks, and after that for the whole sample, without considering fetal ages. To examine whether or not significant differences existed with age, the one-way ANOVA test for unpaired data and then post hoc Bonferroni comparisons were used. The algebraic data for every parameter studied was correlated to fetal age, and linear and nonlinear regression analysis was used to compute the best-fit curve for each parameter considered* versus *gestational age, supported by particular coefficients of determination (*R* ^2^). The relative growth of either lung was expressed as the transverse-to-sagittal diameter ratio, transverse diameter-to-height ratio, and sagittal diameter-to height ratio. Differences were deliberated significant at *p* < 0.05.

## 3. Results

**Figure 3:**
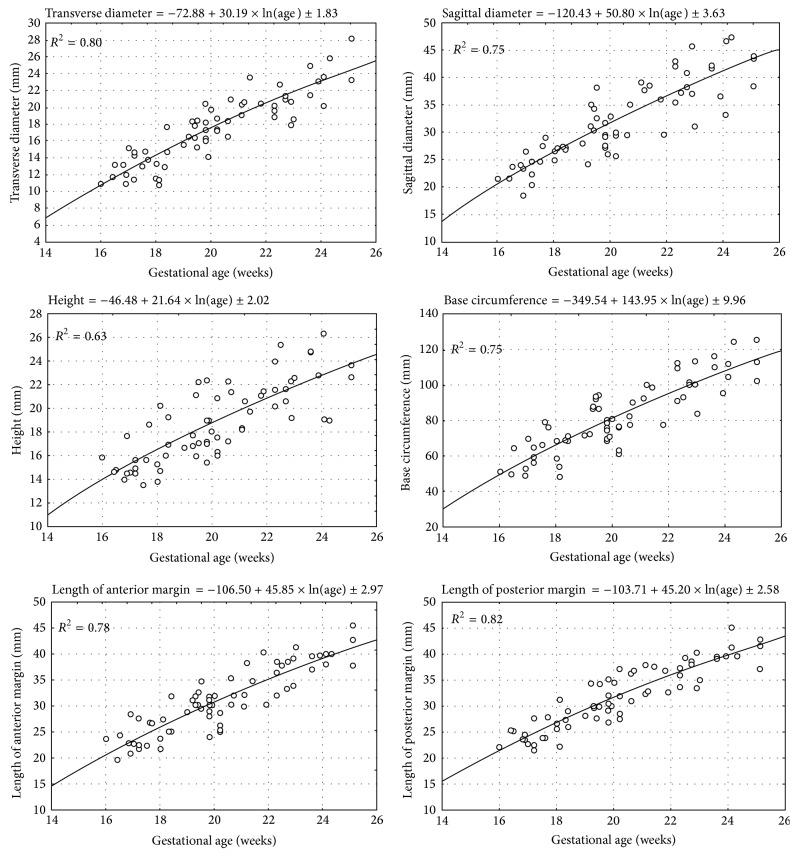
The growth dynamics of the parameters studied of the right lung.

**Figure 4:**
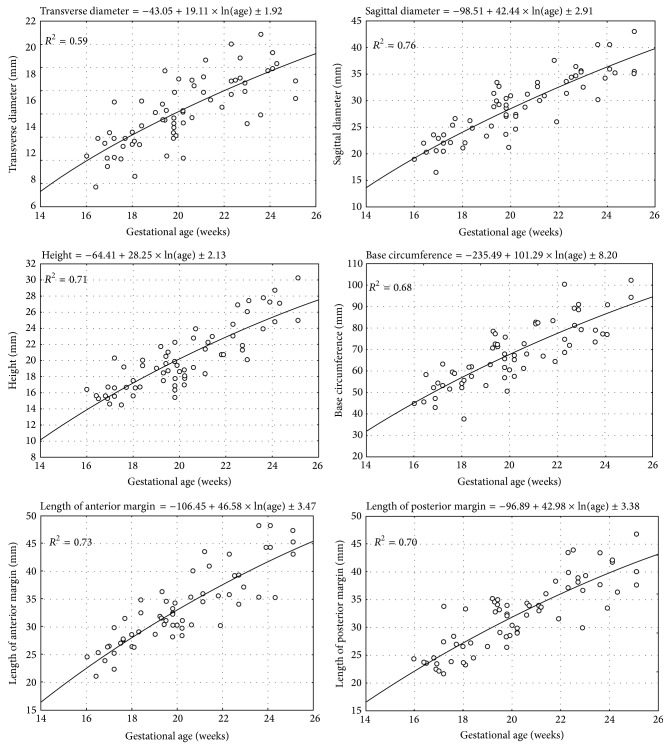
The growth dynamics of the parameters studied of the left lung.

No statistically significant differences (*p* > 0.05) in evaluating intraobserver reproducibility of pulmonary measures were found. The morphometric values obtained were characterized by normality of distribution and homogeneity of variance. As a result, quantitative variables have been expressed as the mean ± standard deviation. Without any significant differences in pulmonary measures with relation to sex (*p* > 0.05), the quantitative data for the right (Table 2) and left (Table 3) lungs for both sexes have been aggregated. On the contrary, there were some laterality differences observed, as follows. Firstly, in the right lungs, their transverse and sagittal diameters and the base circumference were significantly greater (*p* < 0.01) than those in the left ones. Secondly, on the left the anterior and posterior margins and the lung height were significantly (*p* < 0.01) greater than particular parameters on the right. Of note, a statistically significant logarithmic increase in values of all the six measures for the right (Figure 3) and left (Figure 4) lungs was found when related to advancing fetal age. In the right lung, the means for all the pulmonary parameters studied differed significantly at *p* < 0.001 between the three age groups of 16–18, 19–21, and 22–25 weeks. In the left lung, the means for most of the pulmonary parameters studied differed significantly at *p* < 0.001 between the three forenamed age groups, except for the height and length of posterior margin between 16–18 and 19–21 weeks that differed significantly at *p* < 0.01. The growth dynamics of every parameter studied has been displayed in Table 4, including the regression formula of best fit and coefficient of determination (*R* ^2^).

The relative growth of either lung was expressed by the following three indexes: transverse-to-sagittal diameter ratio, transverse diameter-to-height ratio, and sagittal diameter-to-height ratio. The transverse-to-sagittal diameter ratio was stable throughout the analyzed period and averaged 0.56 ± 0.08 and 0.52 ± 0.08 for the right and left lungs, respectively. For the right and left lungs, the transverse diameter-to-height ratio significantly increased from 0.74 ± 0.09 to 0.92 ± 0.08 (*p* < 0.01) and from 0.56 ± 0.07 to 0.79 ± 0.09 (*p* < 0.05), respectively. During the study period, the sagittal diameter-to-height ratio was found to gradually increase from 1.41 ± 0.23 to 1.66 ± 0.18 (*p* < 0.05) in the right lung and from 1.27 ± 0.17 to 1.48 ± 0.22 (*p* < 0.05) in the left lung.

## 4. Discussion

Advances in perinatal medicine result in the early recognition and prompt implementation of corrective procedures in the fetus with life-threatening congenital malformations of the respiratory system [16]. As a prerequisite, a widespread understanding of fetal quantitative anatomy is clearly required so as to produce both normative and pathological criteria adapted to fetal and neonatal respiratory structures [9, 17]. Thus, the current research refers to morphometric analysis of the fetal lungs, providing the existing medical literature with innovative quantitative data. Notwithstanding our findings have been based on 67 human fetuses aged 16–25 weeks; they imitate an age-related sequence in one fetus at the aforementioned age range.

In our opinion, the results achieved in the present study are both normative and factual due to the following three reasons. Firstly, the fetuses studied could be considered normal, because they lacked both external and internal conspicuous anomalies. Besides, they could not suffer from intrauterine growth restriction, since the gestational, amenorrhea, and ultrasound ages proved to be harmonious (*r* = 0.99; *p* < 0.001) [18]. Secondly, since completely degassed fetal lungs perfectly fitted the sealed thoracic cavity, the influence of formalin fixation on lung shrinkage was minimized to roughly 0.5–1.0% [9, 18]. Thirdly, an optimized reliable and objective digital-image analysis system (NIS Elements AR 3.0, Nikon) used in the current study for measuring the clearly defined pulmonary parameters in a direct manner offered real numerical data, instead of deduced, extrapolated through a series of indirect measurements. Of note, digital image analysis proved to be an excellent method of determining the quantitative anatomy of the growing lungs, because all the parameters studied, including the anterior and posterior pulmonary margins and the base circumference, could be perfectly traced using a cursor.

In the material under examination the evidence material covered 12 measurements and 6 calculations for every fetus, resulting in 1206 individual algebraic data for the entire sample. On the other hand, disadvantages of this study may result from both a relatively narrow fetal age (16–25 weeks) and a lack of interobserver variability. Furthermore, it is typical of anatomical research to include only retrospective analysis without prospective ultrasound quality control.

No significant difference in the pulmonary dimensions between two sexes was corroborated in our series. In fact, this remains consistent with all authors, Gerards et al. [19] being excepted, who reported fetal lung volume to be unfettered by sex [20–23]. As reported by Gerards et al. [19], the fetal lung volumes were greater in males by approximately 4.3%. After reviewing the existing literature on laterality differences of the lungs, we managed to find only pulmonary volumetric data, with greater values on the right [9, 12, 19, 21, 23, 24]. It is noteworthy that we found the right-left differences with relation to all the six pulmonary parameters in question. As proved, the transverse and sagittal diameters and the base circumference of the right lungs predominated over the same parameters of the left lungs. In our opinion, these three smaller pulmonary parameters on the left could be restricted by the heart. Furthermore, the three pulmonary features, that is, lengths of anterior and posterior margins, and lung height were considerably smaller on the right, being probably limited by the liver.

In order to choose the best-fit models for the growing lungs, we verified disparate regression formulae from linear to fourth-degree polynomial, taking into account the following three criteria: the greatest *R* ^2^ value, all coefficients different from 0, and the lowest SD of regression [9, 15]. Regrettably, in the estimated second-degree, third-degree, and fourth-degree polynomial models, their parameters were found to be statistically insignificant (*p* > 0.05). Both the linear and logarithmic models were statistically significant (*p* < 0.05 and *p* < 0.001, resp.), but the latter demonstrated greater *R* ^2^ values at the range of 0.59–0.82. Besides, when compared to the linear regressions, the logarithmic models were characterized by the lowest values of both standard deviation for parameters and the standard error of the estimate for the complete model. It is noteworthy that residual value analysis showed normality of distribution for both linear and logarithmic models. In the linear and logarithmic models there were four and two extremal values, respectively, for which standardized residuals were beyond the range of (−2, +2). Finally, the logarithmic models were of best-fit for our empirical data throughout the analyzed fetal period. We substantiated that the growth curves of best-fit for each parameter studied versus gestational ages were natural logarithmic functions, as presented in Table 4. Of note, the greatest *R* ^2^ values referred to the lengths of posterior (*R* ^2^ = 0.82) and anterior (*R* ^2^ = 0.78) margins and transverse diameter (*R* ^2^ = 0.80) of the right lung. The intermediate values of *R* ^2^ were typical of the sagittal diameter and base circumference (*R* ^2^ = 0.75) of the right lung and the sagittal diameter (*R* ^2^ = 0.76), the lengths of anterior (*R* ^2^ = 0.73) and posterior (*R* ^2^ = 0.70) margins, and the height (*R* ^2^ = 0.71) of the left lung. Finally, the lowest *R* ^2^ values characterized the height of the right lung (*R* ^2^ = 0.63) and the remaining two features of the left lung, that is, transverse diameter (*R* ^2^ = 0.59) and base circumference (*R* ^2^ = 0.68). In terms of mathematics, a logarithmic relationship is always one-to-one, continuous, and increasing with a declining rate of change, clearly presented as a concave down graph [15, 17]. This means that an increase in length of the six pulmonary parameters studied gradually decelerated, inexorably deviating downwards from an imaginary axis (*y* = *x*).

Apart from absolute values of the lung dimensions, some novel information on the topic of their relative growth has been addressed by this study. As ascertained, in both lungs the transverse and sagittal diameters evolved proportionately, because the transverse-to-sagittal diameter ratio remained constant for the duration of the study period and attained the values of 0.56 ± 0.08 for the right lung and 0.52 ± 0.08 for the left lung. However, both the transverse and sagittal diameters of either lung grew much faster than the lung height. This fact was meticulously unveiled by the two lung indexes: transverse diameter-to-height ratio and sagittal diameter-to-height ratio. The former considerably increased from 0.74 ± 0.09 to 0.92 ± 0.08 in the right lung and from 0.56 ± 0.07 to 0.79 ± 0.09 in the left lung. The latter gained in values from 1.41 ± 0.23 to 1.66 ± 0.18 and from 1.27 ± 0.17 to 1.48 ± 0.22 for the right and left lungs, respectively.

To the best of our knowledge, the present paper is the first in the medical literature to quantitatively evaluate pulmonary dimensions in question. The complete lack of information in the professional literature relating to the lung parameters studied evidently limits a debate on this subject. The novel growth patterns improve our understanding of pulmonary quantitative morphology and allow calculating the mean of pulmonary parameters according to gestational age. This may particularly be of potential relevance in fetuses suffering from pulmonary hypoplasia, as a result of the following disorders: renal malformations, oligohydramnios, fetal hydrops, skeletal dysplasias, congenital diaphragmatic hernia, intrathoracic masses, congenital adenomatoid malformation, bronchopulmonary sequestration, and cervical and sacrococcygeal teratomata [6, 7]. The medical diagnosis or exclusion of pulmonary hypoplasia may use both prenatal ultrasound and MRI (2–4). In doing so, our quantitative data obtained in this study, as relevant fetal age-specific references for pulmonary parameters, may reliably be conducive. We believe that the normative data for the lung dimensions in the fetus obtained in this study will offer the indispensable background for future autopsy and* in utero *studies.

## 5. Conclusions

The lung dimensions in the fetus divulge no sex differences. The transverse and sagittal diameters and the base circumference are greater in the right lungs, while the lengths of anterior and posterior margins and the lung height are greater in the left lungs. The three-dimensional growth of the fetal lungs follows natural logarithmic functions. In the fetal lungs, their proportionate increase in transverse and sagittal diameters considerably accelerates with relation to the lung height. The lung dimensions in the fetus are relevant in the evaluation of the normative pulmonary growth and the diagnosis of pulmonary hypoplasia.

## Figures and Tables

**Table 1:** Distribution of the fetuses studied.

Fetal age [weeks]^∗^	Crown-rump length [mm]				*n*	Sex	
	Mean	SD	Min	Max		Male	Female
16	111.0	4.2	108.0	114.0	2	1	1
17	122.1	3.7	115.0	126.0	8	4	4
18	136.7	4.3	130.0	142.0	10	5	5
19	153.3	2.0	150.0	155.0	6	4	2
20	161.6	3.3	156.0	166.0	14	7	7
21	174.4	3.8	170.0	180.0	7	2	5
22	188.2	2.5	185.0	190.0	5	3	2
23	195.8	1.8	193.0	198.0	6	4	2
24	208.3	2.9	205.0	212.0	6	4	2
25	220.0	0.0	220.0	220.0	3	1	2

Total					67	35	32

^∗^Note: for anatomists the most objective information for estimating fetal ages is the crown-rump length, when compared to amenorrhea and ultrasound ages.

**Table 2:** Numerical data of the growing right lung.

Age [weeks]	*n*	Right lung (all parameters are expressed in millimeters [mm])											
		Transverse diameter		Sagittal diameter		Height		Base circumference		Anterior margin length		Posterior margin length	
		Mean	SD	Mean	SD	Mean	SD	Mean	SD	Mean	SD	Mean	SD
16	2	11.35	0.62	21.56	0.02	15.28	0.86	50.48	1.08	21.59	2.88	23.79	2.35
17	8	13.14	1.56	22.94	2.53	15.12	1.15	54.77	14.84	23.89	2.75	23.94	1.92
18	10	13.43	2.01	25.57	4.05	16.43	2.31	66.02	9.64	24.64	4.36	26.42	2.67

16–18	20	13.10^(a)^	1.79	24.12^(a)^	3.54	15.79^(a)^	1.87	59.97^(a)^	12.86	24.03^(a)^	3.61	25.17^(a)^	2.59

19	6	17.21	1.17	30.46	4.06	18.62	2.90	84.09	9.71	30.83	1.35	31.32	4.01
20	14	16.90	2.46	29.51	3.98	17.95	2.62	75.50	8.84	29.01	3.05	30.91	3.55
21	7	19.96	2.23	33.89	7.64	19.72	1.86	94.02	12.55	33.33	3.03	34.99	2.83

19–21	27	17.76^(b)^	2.49	31.50^(b)^	4.76	19.18^(b)^	2.78	82.21^(b)^	10.49	30.53^(b)^	2.24	32.06^(b)^	3.79

22	5	18.51	2.99	37.16	5.43	21.70	1.40	92.08	19.42	35.58	4.27	35.36	2.04
23	6	20.37	1.79	38.35	4.81	22.00	2.07	98.93	9.78	37.42	3.14	37.50	2.65
24	6	23.23	2.12	41.23	5.56	22.82	3.11	110.66	9.95	39.14	1.28	40.76	2.26
25	3	23.36	4.80	41.94	3.03	25.22	3.57	113.80	11.68	42.13	3.94	40.55	2.97

22–25	20	21.21^(c)^	3.24	39.45^(c)^	5.02	22.66^(c)^	2.62	102.97^(c)^	14.78	38.19^(c)^	3.63	38.40^(c)^	2.69

Note: between the three age groups of 16–18, 19–21, and 22–25 weeks, the means for all the pulmonary parameters studied marked by letters (a), (b), and (c) in every column differ significantly: (a) *versus* (b) *p* < 0.001, (a) *versus* (c) *p* < 0.001, and (b) *versus* (c) *p* < 0.001.

**Table 3:** Numerical data of the growing left lung.

Age [weeks]	*n*	Left lung (all parameters are expressed in millimeters [mm])											
		Transverse diameter		Sagittal diameter		Height		Base circumference		Anterior margin length		Posterior margin length	
		Mean	SD	Mean	SD	Mean	SD	Mean	SD	Mean	SD	Mean	SD
16	2	9.06	1.88	20.45	2.26	16.07	0.56	45.23	0.49	22.90	2.50	24.12	0.50
17	8	11.60	1.74	21.24	2.35	16.27	1.80	48.83	13.75	25.78	2.18	24.95	4.06
18	10	11.71	1.70	22.84	3.74	17.91	2.40	55.30	7.08	28.08	4.91	28.76	7.80

16–18	20	11.40^(a)^	1.82	21.96^(a)^	3.13	17.07^(a)^	2.16	51.71^(a)^	10.40	26.64^(a)^	4.05	26.77^(a)^	6.26

19	6	15.56	2.52	28.73	3.80	19.54	1.52	69.38	9.61	33.26	4.42	33.09	3.28
20	14	13.28	1.87	27.20	3.47	18.47	1.82	64.75	7.02	30.93	2.23	30.02	2.16
21	7	16.31	1.57	29.80	3.88	21.60	2.09	73.91	8.74	37.26	4.52	33.90	1.20

19–21	27	14.57^(b)^	2.34	28.22^(b)^	3.68	19.52^(b)^	2.21	68.29^(b)^	8.72	33.09^(b)^	4.26	31.71^(b)^	2.83

22	5	17.96	3.25	33.83	5.56	23.59	3.28	78.48	14.38	36.16	4.56	38.09	4.37
23	6	16.46	1.86	34.84	1.31	24.02	3.23	83.78	7.28	37.08	8.10	37.85	4.56
24	6	18.05	2.32	36.14	3.94	26.67	1.84	85.94	16.17	42.64	5.92	39.16	3.87
25	3	20.10	6.94	37.89	4.47	25.37	4.87	78.10	35.32	45.27	2.17	41.54	4.75

22–25	20	17.86^(c)^	3.32	35.44^(c)^	3.88	24.91^(c)^	3.18	82.25^(c)^	16.40	39.75^(c)^	6.67	38.86^(c)^	4.49

Note: between the three age groups of 16–18, 19–21, and 22–25 weeks, the means marked by letters (a), (b), and (c) in every column differ significantly: for transverse diameter: (a) *versus* (b) *p* < 0.001, (a) *versus* (c) *p* < 0.001, and (b) *versus* (c) *p* < 0.001; for sagittal diameter: (a) *versus* (b) *p* < 0.001, (a) *versus* (c) *p* < 0.001, and (b) *versus* (c) *p* < 0.001; for height: (a) *versus* (b) *p* < 0.01, (a) *versus* (c) *p* < 0.001, and (b) *versus* (c) *p* < 0.001; for base circumference: (a) *versus* (b) *p* < 0.001, (a) *versus* (c) *p* < 0.001, and (b) *versus* (c) *p* < 0.001; for length of anterior margin: (a) *versus* (b) *p* < 0.001, (a) *versus* (c) *p* < 0.001, and (b) *versus* (c) *p* < 0.001; for length of posterior margin: (a) *versus* (b) *p* < 0.01, (a) *versus* (c) *p* < 0.001, and (b) *versus* (c) *p* < 0.001.

**Table 4:** The best-fit regression formulae for the growing lungs in the fetus.

Parameter	Right lung		Left lung	
	Regression formula	*R* ^2^ value	Regression formula	*R* ^2^ value
Transverse diameter	*y* = −72.88 + 30.19 × ln⁡(age) ± 1.83	0.80	*y* = −43.05 + 19.11 × ln⁡(age) ± 1.92	0.59
Sagittal diameter	*y* = −120.43 + 50.80 × ln⁡(age) ± 3.63	0.75	*y* = −98.51 + 42.44 × ln⁡(age) ± 2.91	0.76
Height	*y* = −46.48 + 21.64 × ln⁡(age) ± 2.02	0.63	*y* = −64.41 + 28.25 × ln⁡(age) ± 2.13	0.71
Base circumference	*y* = −349.54 + 143.95 × ln⁡(age) ± 9.96	0.75	*y* = −235.49 + 101.29 × ln⁡(age) ± 8.20	0.68
Length of anterior margin	*y* = −106.50 + 45.85 × ln⁡(age) ± 2.97	0.78	*y* = −106.45 + 46.58 × ln⁡(age) ± 3.47	0.73
Length of posterior margin	*y* = −103.71 + 45.20 × ln⁡(age) ± 2.58	0.82	*y* = −96.89 + 42.98 × ln⁡(age) ± 3.38	0.70
